# Exploring crossmodal correspondences for future research in human movement augmentation

**DOI:** 10.3389/fpsyg.2023.1190103

**Published:** 2023-06-15

**Authors:** Mattia Pinardi, Nicola Di Stefano, Giovanni Di Pino, Charles Spence

**Affiliations:** ^1^NeXT Lab, Neurophysiology and Neuroengineering of Human-Technology Interaction Research Unit, Università Campus Bio-Medico di Roma, Rome, Italy; ^2^Institute of Cognitive Sciences and Technologies, National Research Council, Rome, Italy; ^3^Crossmodal Research Laboratory, University of Oxford, Oxford, United Kingdom

**Keywords:** crossmodal correspondence, augmentation, multisensory integration, sensory feedback, embodiment

## Abstract

“Crossmodal correspondences” are the consistent mappings between perceptual dimensions or stimuli from different sensory domains, which have been widely observed in the general population and investigated by experimental psychologists in recent years. At the same time, the emerging field of human movement augmentation (i.e., the enhancement of an individual’s motor abilities by means of artificial devices) has been struggling with the question of how to relay supplementary information concerning the state of the artificial device and its interaction with the environment to the user, which may help the latter to control the device more effectively. To date, this challenge has not been explicitly addressed by capitalizing on our emerging knowledge concerning crossmodal correspondences, despite these being tightly related to multisensory integration. In this perspective paper, we introduce some of the latest research findings on the crossmodal correspondences and their potential role in human augmentation. We then consider three ways in which the former might impact the latter, and the feasibility of this process. First, crossmodal correspondences, given the documented effect on attentional processing, might facilitate the integration of device status information (e.g., concerning position) coming from different sensory modalities (e.g., haptic and visual), thus increasing their usefulness for motor control and embodiment. Second, by capitalizing on their widespread and seemingly spontaneous nature, crossmodal correspondences might be exploited to reduce the cognitive burden caused by additional sensory inputs and the time required for the human brain to adapt the representation of the body to the presence of the artificial device. Third, to accomplish the first two points, the benefits of crossmodal correspondences should be maintained even after sensory substitution, a strategy commonly used when implementing supplementary feedback.

## 1. Introduction

### 1.1. Crossmodal correspondences

“Crossmodal correspondences” are the consistent matchings between perceptual dimensions or stimulus attributes from different sensory domains that are observed in normal observers (i.e., in non-synesthetes; see Spence, 2011, for a review). One of the most famous audiovisual associations was discovered almost a century ago by Köhler (1929). In particular, the German psychologist observed that people tend to associate the term “baluba” with curved lines, while the term “takete” is associated with angular shapes instead. To date, the existence of a very wide range of such crossmodal associations have been demonstrated, involving most, if not all, combinations of senses (see Spence, 2011, for a review). Associations involving auditory and visual stimuli have been predominantly addressed. That said, combinations involving haptics (Slobodenyuk et al., 2015; Wright et al., 2017; Lin et al., 2021) and/or the chemical senses (i.e., taste and gustation) have increasingly been investigated over the last decade, also in relation to the growing interest in research on food and wine pairing, and food marketing/packaging design (North, 2012; Spence and Wang, 2015; Pramudya et al., 2020; Spence, 2020).

Crossmodal associations have primarily been investigated in the field of experimental psychology/perception research, in which it has, on occasion, been related to the wider phenomenon of multisensory integration (Parise and Spence, 2009), conceived of as the processing and organization of sensory inputs received from different pathways into a unified whole (Tong et al., 2020). A number of intriguing questions concerning the nature of crossmodal correspondences and the relative contribution of different factors remains open, with some researchers putting forward the existence of intersensory, or suprasensory stimulus qualities, such as brightness, intensity, roughness, that can be picked-up by multiple senses (e.g., see, von Hornbostel, 1931; Walker-Andrews et al., 1994), and others focusing more on the integration of information occurring in the brain, as a result of crossmodal binding mechanisms (Calvert and Thesen, 2004).

### 1.2. Multisensory processing and feedback in the context of human augmentation

The mechanisms behind the processing of multisensory stimuli are also of great interest to scientists seeking feedback strategies to be implemented in the context of human motor control. Indeed, multisensory feedback has been shown to improve an individual’s ability to command their body significantly, especially in complex interactions requiring constant adjustment and recalibration (Wolpert et al., 1995; Diedrichsen et al., 2010; Shadmehr et al., 2010). The same benefit can potentially also be extended to the control of artificial devices, such as teleoperated robots or prostheses. In the field of prosthetics research, researchers have endowed prostheses with artificial senses, so that users can retrieve sensory information from these artificial devices as they would with their natural limbs, ultimately leading to better control and higher acceptability (Di Pino et al., 2009; Di Pino et al., 2012, 2020; Raspopovic et al., 2014; Marasco et al., 2018; Page et al., 2018; Zollo et al., 2019).

In recent years, the role of multisensory feedback has been studied within the framework of human augmentation, an emerging field that aims at enhancing human abilities beyond the level that is typically attainable by able-bodied users (Di Pino et al., 2014; Eden et al., 2021). Movement augmentation can, for instance, be achieved through the use of supernumerary robotic limbs (SRLs), artificial devices that can be controlled simultaneously with, but independently from, the natural limbs, thus opening up new possibilities in the interaction with the environment. SRLs can be controlled through the movement of body parts that may not be directly involved in the task, actuated by means of joysticks, trackers, or retrieved from the electrical muscle activity (Eden et al., 2021). It turns out that the quality of the motor interface is of pivotal importance as far as achieving proficient performance is concerned. However, proficient control can only be achieved by closing the sensorimotor loop between user and robotic device by means of reliable feedback, possibly covering multiple senses. Indeed, it has recently been demonstrated how supplementary sensory feedback improved the regulation of the force exerted by the SRL’s end-effector when interacting with an object (Hussain et al., 2015; Hernandez et al., 2019; Guggenheim and Asada, 2021), as well as increasing the accuracy in reaching a target or replicating the end-effector position (Segura Meraz et al., 2017; Aoyama et al., 2019; Pinardi et al., 2021, 2023). It can also reduce the amount of time required to complete behavioral tasks (Hussain et al., 2015; Sobajima et al., 2016; Noccaro et al., 2020).

Multisensory integration plays a key role in building body representations, the map that our brain uses to recognize and identify our body and the model the brain uses to control its movement (i.e., body image and body schema; de Vignemont, 2010; Blanke, 2012; Moseley et al., 2012). Sensory feedback continuously updates these representations, allowing the brain to accept artificial limbs, as has frequently been demonstrated in the famous Rubber Hand Illusion (Botvinick and Cohen, 1998), where a visible rubber hand and the participant’s own hidden hand are brushed synchronously (though see Pavani et al., 2000, for evidence that synchronous stroking is not always required to induce the illusion). As a result of this visuotactile congruency, the artificial limb is included in the body representation and thus perceived as belonging to the body of the person experiencing the illusion. This process, often labeled “embodiment” (Botvinick and Cohen, 1998; Pinardi et al., 2020a), has been reported for several types of artificial limbs, including prostheses, and has been linked to an improvement in artificial limb control and acceptability (Marasco et al., 2011; Gouzien et al., 2017). Recent works suggests that embodiment might be perceived for SRLs as well, making it a relevant topic in the field of human augmentation (Di Pino et al., 2014; Arai et al., 2022; Di Stefano et al., 2022; Umezawa et al., 2022).

### 1.3. Perspectives for future research in human augmentation

**FIGURE 1:**
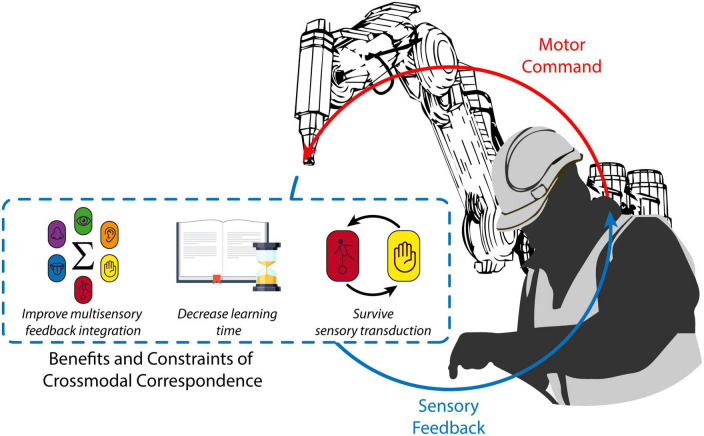
Crossmodal correspondences relevant to human augmentation. Relying on crossmodal correspondences when designing the SRL supplementary feedback encoding (blue dotted line) could make a robot status parameter (e.g., its position) relayed through multiple sensory modalities (e.g., somatosensation and vision), more useful and easier to integrate. This, in turn, might help to decrease the amount of time required to learn to efficiently decode SRL feedback. Existing evidence suggests that these benefits should be resistant to sensory modality transduction. As a result, supplementary sensory feedback from the SRL (solid blue line) should be more efficient and motor control (solid red line) and embodiment of the augmenting device should improve.

In this paper, we present three novel considerations concerning how future research on human augmentation might benefit from the vast knowledge concerning crossmodal correspondences. First, crossmodal correspondences could make multisensory feedback from the augmenting device easier to process and understand. Second, the spontaneous and widespread mechanisms that characterize crossmodal correspondences might well be expected to reduce the amount of time that is needed for users to learn and decode the supplementary feedback of SRLs and to embody it faster. Third, the benefits of crossmodal correspondences involving two sensory modalities should persist if one modality is transduced but the informative content is maintained (see Figure 1).

## 2. Improving supplementary feedback integration

Crossmodal correspondences modulate multisensory integration (see Spence, 2011, for a review). More specifically, crossmodally congruent multisensory stimuli possess a perceptual bond that leads to increased strength/likelihood of integration thus potentially reducing spatiotemporal discrepancy. Indeed, when participants had to judge the temporal order of auditory and visual stimuli presented in pairs, worse performance was observed with pairs of stimuli that were crossmodally congruent as compared to those that were incongruent (Parise and Spence, 2009).

Crossmodally congruent pairs of audiovisual stimuli give rise to larger spatial ventriloquism effects (i.e., mislocalization of the source of a sound in space toward an irrelevant visual stimulus) compared to incongruent pairs (Parise and Spence, 2008). Crossmodal correspondences can also impact the perceived direction of moving visual stimuli: participants perceived gratings with ambiguous motion as moving upward when coupled with an ascending pitch sound and vice versa (Maeda et al., 2004).

Additional research findings have also demonstrated that crossmodal correspondences can improve behavioral performance in sensorimotor tasks. For instance, in a speeded target discrimination task in which participants were asked whether the pairs of color and sound stimuli that were presented appropriately matched, higher accuracy and faster reaction times were obtained with crossmodally congruent pairs of stimuli as compared to incongruent ones (Marks, 1987; Klapetek et al., 2012; Sun et al., 2018). Recently, audiovisual correspondences were shown to promote higher accuracy and faster responses during a reaction time task when stimuli were congruent rather than incongruent (Ihalainen et al., 2023). Even complex motor tasks, such as drawing, are affected by crossmodal correspondences, with participants obtaining higher accuracy in representing graphically certain features of auditory stimuli (e.g., pitch) compared to others (e.g., loudness) (Küssner and Leech-Wilkinson, 2014). Therefore, a first suggestion is that crossmodal correspondences can be exploited to improve the integration of multisensory information concerning the status of the SRL. According to the Bayesian integration framework (Körding and Wolpert, 2004; Chandrasekaran, 2017), supplementary feedback is particularly useful when it carries information with lower “noise” (i.e., less disturbance in the signal) compared to other sensory cues. Hence, if the user is operating the SRL in an environment with reduced visibility (e.g., presence of smoke or dust, or darkness for workers operating under water), supplementary feedback carrying information on the motion of the robot would become particularly useful. This usefulness could be increased even more by capitalizing on the correspondence between perceived motion and acoustic pitch. For instance, playing an ascending pitch when the robot moves upward would easily bias the attention of the user toward that direction, because of the integration guided by crossmodal correspondence, thus improving the interaction under low visibility conditions.

## 3. Reducing learning time

A striking feature of crossmodal correspondences is how spontaneous and widespread they appear to be. Whether this perceptual phenomenon reflects an innate and universal mechanism is not yet clear, since research has produced conflicting evidence (Ludwig et al., 2011; Spence and Deroy, 2012). Despite an experience-based explanation for certain crossmodal correspondences having been proposed (Speed et al., 2021), and possible cultural differences between groups occasionally being reported (Spence, 2022), the empirical evidence that has been published to date shows that most healthy adults perceive crossmodal correspondence spontaneously, consistently, and without the need for any specific training (though see also Klapetek et al., 2012). The possibility of associating information from different sensory modalities without a dedicated training is of great interest for the field of human augmentation.

Indeed, learning to use a sensory augmentation tool requires the user to become familiar with its physical features (e.g., dimensions, weight, joint stiffness, and degrees of freedom) in order to interact with it proficiently. Rich supplementary feedback can greatly facilitate this process, but learning to understand and use such feedback often requires extensive training, especially when supplementary feedback carries information that is not available to the other senses. This sensory substitution requires being exposed to both the original sensory feedback (e.g., vision) and the supplementary one (e.g., vibrotactile stimulation) in order to establish an association that is sufficiently strong to survive the removal of the former and thus allow the latter to provide useful information. The latest research demonstrates that this is possible in the specific context of human augmentation (Pinardi et al., 2021; Umezawa et al., 2022). However the long training sessions required to fully benefit from supplementary feedback, can all too easily result in participant exhaustion and demotivation, thus limiting the feasibility of the experimental approach. To avoid this, supplementary feedback usually relies on a simple encoding strategy that can be learned quickly. For instance, it is useful to respect the spatial distribution between workspace organization and feedback interface on the participant’s body (i.e., feedback of proximal joints is delivered proximally on the participant’s body; Noccaro et al., 2020; Pinardi et al., 2021), or to couple a vibrotactile stimulation frequency that is higher with a higher force exerted by the robotic limb (Hussain et al., 2015). By removing the need for feedback decoding, the effort to facilitate feedback learning could be pushed toward a more efficient application by exploiting the spontaneous associations that characterize crossmodal correspondences, such as the well-documented space-pitch/loudness correspondence.

### 3.1. Application scenario: exploiting space-pitch/loudness correspondence for SRL localization

Pitch is consistently associated with spatial locations on the vertical (Bordeau et al., 2023), and to a lesser extent horizontal, axis, such that a low-pitched sound is more likely to be associated with a left/low location whereas a high pitch is associated with a right/high location (Mudd, 1963; Rusconi et al., 2006; Spence, 2011; Guilbert, 2020). At the same time, however, loudness is known to be an effective indicator of distance, such that, when presented with two sounds which differ only in loudness, people tend to associate the louder sound with a closer sound source, and *vice versa* (Eitan, 2013; Di Stefano, 2022).

**FIGURE 2:**
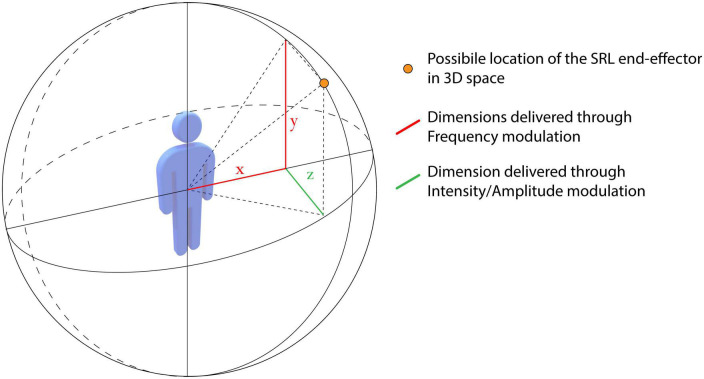
Possible encoding strategy that exploit visuo/haptic/auditory crossmodal correspondences to facilitate the localization of an SRL end-effector. 3D space is represented as a sphere surrounding the user, while the possible location of the SRL end-effector is shown as an orange dot. Dimensions that could be delivered by modulating sound (or possibly vibrotactile) frequency are shown in red. Since pitch modulation has been associated with localization on both the *x* and, especially, *y* axis (red), disentangling them while using the same encoding strategy (i.e., frequency modulation) remains an open issue that could potentially be addressed initially by simplifying the scenario and considering only the *y* axis. Dimension that could be delivered by modulating either sound intensity or possibly vibrotactile amplitude is shown in green.

Sonification is a strategy of heteromodal sensory substitution which relays kinematic and dynamic features of a movement with sounds (Castro et al., 2021a,b); however, its effectiveness is dependent on the specific rules used for encoding. Sonification can also be exploited to relay feedback related to an SRL. In light of enhancing the intuitiveness and hence usability of sensory substitution devices by means of the incorporation of crossmodal correspondence, we propose to use sound frequency and intensity (which determines perceived pitch and loudness, respectively) to help users to more easily localize the SRL in tri-dimensional space with minimal need for dedicated training, since pitch/space correspondence has been shown to occur spontaneously (Guilbert, 2020), even in new-borns (Walker et al., 2018), and space/loudness is a reliable and consistent association which reflects the regularities in the environment (Eitan, 2013) (see Figure 2). Given that the spatial resolution of pitch/space correspondences has yet to be determined, it is hard to provide clear indications on the effective contribution of this association to the spatial localization of SRLs. However, a rough indication on the region of space occupied by the SRL in terms of the two principal axes (i.e., up/down, and right/left) would be probably enough informative for these kinds of tasks, and we expect that such information can be likely provided intuitively, thus potentially reducing the learning time.

The association between pitch and the vertical/horizontal axes could also be used in human augmentation to generate more “instinctive” warning signals that might direct the operators’ spatial attention in an intuitive way, i.e., with no need for dedicated training (Ho and Spence, 2008). Finally, sonification has been shown to improve the behavioral performance of sportsmen (e.g., Maes et al., 2016; Lorenzoni et al., 2019; O’Brien et al., 2020), and thus coupling it with crossmodal correspondence mechanisms might further facilitate this beneficial effect. Since pitch is associated at the same time with both horizontal and vertical direction, it would be important to disentangle the modulation of these axis. This could be addressed initially by simply considering one of the two axes (2D workspace), and later by acting on how feedback is delivered (e.g., deliver two pitches sequentially, the first coding the *x* axis and the second coding the *y* axis). Additionally, while the loudness/distance association has been investigated primarily on the *z* axis, it should be tested whether it also consistently present on the others.

This perspective is further corroborated by studies involving blind participants, that show that the use of sensory substitution devices, despite not being devoid of challenges (Barilari et al., 2018; Hamilton-Fletcher et al., 2018; Sourav et al., 2019), is somewhat easier to learn if they are based on crossmodal correspondences (Hamilton-Fletcher et al., 2016) and a similar correspondence-based facilitation has also emerged in the case of language learning as well (Imai et al., 2008, 2015).

Moreover, it can be predicted that the haptic feedback might be used similarly to pitch, assuming that frequency and amplitude of a vibrotactile stimulation are associated with vertical/horizontal localization and with distance, or depth, respectively. Should this be confirmed empirically, it would represent a useful principle to guide the design of supplementary feedback. Additionally, given the multifaceted nature of the somatosensory modality, haptic feedback could potentially convey several informative contents simultaneously, so that multiple stimulus dimensions can be captured more clearly compared to information conveyed through auditory feedback. Indeed, somatosensation includes a great variety of sensory stimuli, such as, vibration, pressure, and skin stretch. Combining different types of stimuli, such as high frequency vibrotactile stimulation and skin stretch, to encode different information (e.g., coordinates on different axis), could reduce the risk of ambiguous encoding while maintaining a high informative value. Though, that being said, one should never forget the inherent limitations associated with the skin as a means of information transfer (see Spence, 2014).

## 4. Sensory transduction

Studies on human motor control have proven that proprioception is an unobtrusive yet rich sensory modality allowing people to complete dextrous motor tasks with minimal attentional effort (Proske and Gandevia, 2012). This is further confirmed in patients who lost proprioception, and must continuously rely on visual monitoring to carry out daily life actions, with a huge attentional effort and lower learning performance (Danna and Velay, 2017; Miall et al., 2019, 2021). Hence, relaying proprioceptive-like information concerning SRLs (i.e., joint angle, end effector acceleration or exerted force) to the user can result in improved SRL control. However, in the case of supplementary feedback, it is often difficult to adopt a homomodal coding approach (information related to a given sensory modality are fed back to the user exploiting the same sensory modality e.g., pressure on the robot surface with tactors upon the user skin). This is due to the fact that SRL’s proprioceptive parameters should be delivered using proprioceptive stimulation, and this is sometimes impossible or impractical (e.g., delivering joint angles by means of a kinesthetic illusion (Pinardi et al., 2020b) imposes heavy constraints on experimental protocols). Hence, translating a proprioceptive parameter of the robot (e.g., joint angle) into a pattern of tactile stimulation (e.g., vibratory stimulation) is easier to implement, resulting in heteromodal feedback. This approach solves a number of technical issues but introduces the need to translate supplementary sensory feedback from one modality (e.g., proprioception) to another (e.g., touch). Hence, the possibility of maintaining the benefits of crossmodal correspondence across different sensory modalities would be an empirical direction worth investigating. For instance, higher auditory pitch is associated with faster motion (Zhang et al., 2021) and higher physical acceleration (Eitan and Granot, 2006). However, in a human augmentation paradigm, participants would not be directly informed about speed or acceleration through supplementary feedback, but rather with vibratory stimulation that codes those parameters (e.g., higher vibrotactile frequency codes for higher acceleration). Hence, would the association between acceleration (or speed) and auditory pitch still be valid, if acceleration were to be substituted with a vibrotactile stimulation coding the same information?

To the best of our knowledge, there are no studies that directly address this question, however, considering that crossmodal correspondences have been demonstrated between every pair of sensory modalities (North, 2012; Nava et al., 2016; Wright et al., 2017; Speed et al., 2021) and are, by definition, cross-modal, it is likely that the underlying mechanisms would not be disrupted by sensory modality transduction.

## 5. Discussion and conclusion

In the present perspective paper, possible intersections between two different and, to the best of our knowledge, previously unrelated research fields, namely human augmentation and crossmodal correspondences have been explored. We propose that future research on the control and embodiment of SRLs could benefit from exploiting the spontaneous and widespread mechanisms of crossmodal correspondences, which have been shown to impact on spatiotemporal features and constraints of multisensory integration. The potential improvement of the control of SRLs would likely result as a by-product of the well-documented positive effect of crossmodal correspondence on attentional processing (Spence, 2011; Klapetek et al., 2012; Orchard-Mills et al., 2013; Chiou and Rich, 2015). Indeed, attention is considered a crucial resource when it comes to learning new motor skills and improving motor performance, especially in complex environments or during the performance of difficult tasks (Song, 2019), such as controlling an SRL. The link between motor control, attention and sensory processing is further demonstrated by the fact that multisensory contingencies can modulate the coupling between attention and motor planning (Dignath et al., 2019). However, further evidence is required to assess and disentangle the specific impact of crossmodal correspondences on motor control or attentional processing in the framework of human augmentation.

While the insights suggested here could be extended to any robotic teleoperated device, they are particularly fit for the field of human augmentation. Indeed, SRL users are supposed to control robotic limbs and natural ones at the same, while receiving feedback from both. This produces an additional challenge that is not present in prosthetics or when dealing with robotic teleoperation. The added sensorimotor processing required in human augmentation, and the absence of residual neural resources that can be repurposed for conveying feedback (Makin et al., 2017; Dominijanni et al., 2021) justifies the need for new strategies that minimize the effort required for sensory feedback learning and maximize its usefulness, and even though benefits of crossmodal correspondence have usually been observed in simple tasks (i.e., speeded target discrimination) and control and embodiment of an augmenting device are much more complex tasks, they ultimately rely on the same mechanisms of multisensory integration.

Finally, we argue that crossmodal correspondences could be exploited to deliver even highly sophisticated feedback with reduced cognitive burden. For instance, by capitalizing on the correspondence between tactile and auditory roughness (see Di Stefano and Spence, 2022, for a review), it might be possible to deliver the sensation of a rough texture through a rough sound (e.g., equal intensity for all frequencies, such as white noise, Pellegrino et al., 2022). More specifically, simply using a camera located on the tip of the end-effector, it would be possible to analyze surface texture, determine its roughness level through a machine-learning algorithm, and modulate the features of an auditory cue to provide information to the user on the surface tactile quality (Guest et al., 2002).

## Data availability statement

The original contributions presented in this study are included in the article/supplementary material, further inquiries can be directed to the corresponding author.

## Author contributions

MP and NDS conceived the work and wrote the first draft of the manuscript. CS and GDP revised the entire manuscript and provided the insightful comments on all sections. All authors read and approved the submitted version.
